# The effect of precooling on short term performance

**DOI:** 10.1186/2046-7648-4-S1-A124

**Published:** 2015-09-14

**Authors:** Panagiotis Gkrilias, Athanasios Zavvos, Niki Manolaki, Nikolaos Geladas, Maria Koskolou, Spyros Athanasopoulos

**Affiliations:** 1 Department of Sports Medicine and Biology of Exercise, Faculty of Physical Education and Sport Science, University of Athens, Greece; 2 Technological Educational Institute (TEI) of Western Greece, Department of Physical Therapy, Aigio Achaias, Greece

## Introduction

The benefits of precooling have been widely studied and are mainly attributed to a smaller rise of body core temperature (Tc) and improved cardiovascular responses in prolonged exercise [1], [2]. Regarding the performance of short-term high-intensity exercise, after precooling, the results are conflicting [3]. The purpose of this study was to examine whether short-term performance could be affected by whole body immersion (at chest level with arms and hands out of the water) for 30 minutes at 16 °C to 18 °C.

## Methods

On two occasions, 10 male volunteers {age: 23.2 (2) yr, height: 178,1 (7.1) cm, body mass: 77.6 (6.4) kg, body fat: 12 (2.7) %, VO_2max_: 44.3 (4.9) mL.Kg^-1^.min^-1^} performed an intermittent exercise bout (Cisp), consisting of five repeated 5-sec sprints on a mechanical cycle ergometer [4] and, subsequently, a short high-intensity shuttle-run test (6 × 5 meters) either or without precooling (Cool Vs Con) in random and counterbalanced order. The Cisp was performed in an environmental chamber (temperature: 31 °C to 33 °C, rh 40% to 50%) while, for technical reasons the shuttle-run test was performed in thermoneutral environment (19 °C to 22 °C). Performance was evaluated as the peak power output per sprint (Pmax) and the total time to complete the shuttle-run test. In specific parts of the protocol rectal temperature (T_re_), the temperature of the finger (Tfin) and forearm (Tfarm), heart rate (Hr), thermal sensation (Ts) and perceived fatigue (Borg scale) were recorded.

## Results

A reduction (p ≤ 0.001) in Pmax {Pmax 1^st ^sprint (0-2 min): Cool: 806.7 (63.1) Watt Vs Con: 860.9 (78.7) Watt, Pmax 5^th ^sprint (8-10 min): Cool: 856.8 (74.7) Vs Con: 912.8 (70.4) Watt} and a tendency (p = 0.06) for longer time to complete the shuttle-run test were observed in Cool {10.43 (0.66) sec} compared to Con {10.18 (0.41) sec} condition. The T_re _during the Cisp test in Cool condition {Tre 0-2 min Cool: 37,10 (0.72) °C, Tre 8-10 min Cool: 37,35 (0.77) °C}, was lower (p ≤ 0.05), compared with Con values {Tre 0-2 min Con: 37.54 (0.25) °C, Tre 8-10 min Con: 37.84 (0.18) °C}. The vasoconstriction index (Tfarm-Tfin) [5], was higher (p ≤ 0.001) throughout the duration of the exercise protocol in Cool than in the Con condition {Index 0-2 min: Cool: 5.56 (0.76) °C Vs Con: 0.66 (2.15) °C, Index 8-10 min: Cool: 3.94 (2.09) °C Vs Con: -0.63 (1.63) °C}.

## Discussion

After precooling with whole body immersion a reduction in short term performance was observed which was probably due to neuromuscular system dysfunction caused by coolness.

## Conclusion

This type of information, about the short term performance reduction after precooling, observed in our study, should be taken into consideration by the technical and medical staff of athletic teams, in sports which demand explosiveness and somatic contact right after precooling.
